# Knockdown of *EIF4G1* in NSCLC induces CXCL8 secretion

**DOI:** 10.3389/fphar.2024.1346383

**Published:** 2024-02-09

**Authors:** Ziyang He, Fangyi Li, Xinyi Zhang, Dacheng Gao, Zhiwen Zhang, Rui Xu, Xingguo Cao, Qiyuan Shan, Zhen Ren, Yali Liu, Zengguang Xu

**Affiliations:** ^1^ Research Center for Translational Medicine, Shanghai East Hospital, School of Medicine, Tongji University, Shanghai, China; ^2^ Shanghai East Hospital, Postgraduate Training Base of Jinzhou Medical University, Shanghai, China

**Keywords:** NSCLC, EIF4G1, CXCL8, IL8, chemotaxis, MAPK

## Abstract

Non-small cell lung cancer (NSCLC) is the most common type of lung tumor; however, we lack effective early detection indicators and therapeutic targets. Eukaryotic translation initiation factor 4 gamma 1 (EIF4G1) is vital to initiate protein synthesis, acting as a scaffolding protein for the eukaryotic protein translation initiation factor complex, EIF4F, which regulates protein synthesis together with EIF4A, EIF4E, and other translation initiation factors. However, EIF4G1’s function in NSCLC cancer is unclear. Herein, transcriptome sequencing showed that knockdown of *EIF4G1* in H1299 NSCLC cells upregulated the expression of various inflammation-related factors. Inflammatory cytokines were also significantly overexpressed in NSCLC tumor tissues, among which *CXCL8* (encoding C-X-C motif chemokine ligand 8) showed the most significant changes in both in the transcriptome sequencing data and tumor tissues. We revealed that EIF4G1 regulates the protein level of TNF receptor superfamily member 10a (TNFRSF10A) resulting in activation of the mitogen activated protein kinase (MAPK) and nuclear factor kappa B (NFκB) pathways, which induces CXCL8 secretion, leading to targeted chemotaxis of immune cells. We verified that H1299 cells with *EIF4G1* knockdown showed increased chemotaxis compared with the control group and promoted increased chemotaxis of macrophages. These data suggested that EIF4G1 is an important molecule in the inflammatory response of cancer tissues in NSCLC.

## Introduction

Lung tumors are a major threat to global health. According to tumor statistics, nearly 2 million people worldwide are diagnosed with lung cancer each year (Siegel et al., 2022). According to histological classification, lung cancer is mainly divided into two categories: small cell lung cancer and non-small cell lung cancer (NSCLC), among which NSCLC is subdivided into squamous cell carcinoma, adenocarcinoma, and large cell lung cancer (Juretic et al., 1999). NSCLC accounts for 80%–85% of lung tumors, and is characterized by difficulties in early detection, an inability to be eradicated by surgical treatment, and ineffectiveness of radiotherapy and chemotherapy, which seriously endangers human health (Stower, 2020; Chen et al., 2022; Li Y. et al., 2023b). Currently, it is difficult to improve the 5-year survival rate of NSCLC by improving traditional treatment strategies (surgery and radiotherapy). Recently, research in translational medicine has led to the discovery of new tumor detection methods and targets, and some targeted drugs have been developed. However, the pathogenesis of NSCLC is unclear and there is a lack of effective early detection indicators, such that three-quarters of lung cancer patients are diagnosed with Stage III or Stage IV NSCLC, by which time the disease has spread to the lymph nodes or other organs and cannot be cured (Kalinke et al., 2021).

Eukaryotic translation initiation factor 4 gamma (EIF4G) is a key component in the initiation of protein synthesis, and has three isoforms: EIF4G1, EIF4G2, and p97/NAT1/DAP-5, among which EIF4G1 is an important isoform. EIF4G1 acts as a scaffolding protein for the eukaryotic protein translation initiation factor complex EIF4F, which comprises the cap-binding protein eIF4E, the DEAD-box RNA helicase eIF4A (which also exists in a free form), and the eIF4G scaffold, to regulate protein synthesis (Bellsolell et al., 2006; Tu et al., 2010; Liu et al., 2022; Brito Querido et al., 2023). A recent study showed that EIF4G1 is associated with tumorigenesis and progression. It was reported that the MET proto-oncogene, receptor tyrosine kinase (MET), regulates the expression level of hypoxia-inducible factor 1 alpha (HIF-1α) through a protein translation mechanism regulated by phosphorylation of EIF4G1 on serine 1,232 and showed that it has an impact on the disease-free survival of patients with NSCLC (Glück et al., 2018). *EIF4G1* might be a mesothelial cancer driver gene, and its upregulation could play a part in mesothelial cell tumorigenesis and aggressiveness (Dell'Anno et al., 2020). Another report showed that EIF4G1 could be a biomarker for the prognosis of patients with nasopharyngeal carcinoma. The authors found that the expression level of *EIF4G1* mRNA in nasopharyngeal carcinoma tissues and cell lines was significantly higher than that in normal nasopharyngeal tissues and NP69 cells. Further multivariate analysis showed that *EIF4G1* expression was an independent prognostic indicator for overall survival in patients with nasopharyngeal carcinoma; indeed, knocking down *EIF4G1* using a short hairpin RNA (shRNA) not only significantly inhibited cell cycle progression, proliferation, migration, invasion, and colony formation, but also greatly inhibited the growth of xenograft tumors *in vivo* (Tu et al., 2010).

Inflammation in the tumor microenvironment (TME) has an important role in neoplasm development (Zhao et al., 2017; Li et al., 2021). Under normal circumstances, such as wound healing, the influx of cytokines and chemokines heals the wound in a self-limiting manner. However, dysregulation of this process might lead to an abnormal inflammatory response, ultimately leading to tumorigenesis. Tumor cells themselves produce cytokines that attract neutrophils, macrophages, lymphocytes, and dendritic cells, and the presence of these cells in the TME has been shown to be pro-tumorigenic and correlate with a poorer disease prognosis in animal models and patients with cancer (Teijeira et al., 2021; Xiong et al., 2021; Mantovani et al., 2022; van Vlerken-Ysla et al., 2023). By contrast, C-X-C motif chemokine ligand 8 (CXCL8), is an important molecule among the inflammation-related cytokines, playing many vital roles in tumors, including promoting the proliferation of pancreatic cancer cells, affecting the migration and invasion of melanoma, promoting tumor angiogenesis and affecting the tumor immune microenvironment via chemotaxis of neutrophils, macrophages, and endothelial cells (Vandercappellen et al., 2008; Legler et al., 2018). Knockdown of *CXCL8* inhibited angiogenesis and tumor growth and CXCL8 might serve as a potential new biomarker and therapeutic target to overcome epidermal growth factor (EGRF)-tyrosine kinase inhibitor (KI) resistance in the future (Liu et al., 2023). Recently, the chemokine CXCL8 has been discovered to play an important role in tumor progression. CXCL8 activates multiple intracellular signaling pathways by binding to its receptor (CXCR1/2), and plays two-fold pro-tumorigenic roles in the TME, including the direct promotion of tumor survival and the indirect promotion of tumor progression by influencing the components of the TME, which include promotion of tumor cell proliferation and epithelial-to-mesenchymal transition (EMT), promoting angiogenesis and inhibiting anti-tumor immunity (Cambier et al., 2023). The most important role of CXCL8 as a chemokine is its pro-inflammatory function in immune cells. Upon microbial exposure, macrophages release large amounts of CXCL8, thereby promoting the chemotaxis of immune cells to migrate to the site of antigenic attack (Mantovani et al., 2022).

In this study, we screened CXCL8, a effector molecule downstream of EIF4G1, by transcriptome sequencing and analyzing clinical samples. We verified the sequencing results at the mRNA level and protein levels. However, EIF4G1 could not increase the mRNA level and the protein levels of CXCL8 at the same time, and we identified an intermediate factor between EIF4G1 and CXCL8, TNF receptor superfamily member 10a (TNFRSF10A). TNFRSF10A increased the phosphorylation of extracellular regulated kinase 1/2 (ERK1/2) and JUN N-terminal kinase (JNK)1/2/3 in the mitogen activated protein kinase (MAPK) pathway, and the nuclear factor kappa B (NFκB) inhibitor alpha (IκBα) in the NFκB pathway, leading to the upregulation of CXCL8 expression. Finally, we verified that CXCL8 promoted macrophage chemotaxis, which provided new pathways for diagnostic and immunotherapy targeted research in NSCLC.

## Materials and methods

### Cell culture

Human lung cancer cells H1299 were obtained from the Chinese Academy of Sciences and were cultured in Dulbecco’s modified Eagle’s medium (DMEM; Corning Inc., Corning, NY, United States). Human monocytic leukemia cells, THP-1 (iCell Bioscience Inc., Shanghai, China), were cultured in Roswell Park Memorial Institute (RPMI 1640 medium (Corning). Cell culture was performed with 10% fetal bovine serum (Corning) and 1% penicillin streptomycin (Corning). Cells were cultured at 37°C, 5% CO_2_, and 95% humidity in an incubator (Thermo Fisher Scientific, Waltham, MA, United States).

### RNA sequencing (RNA-seq) and quantitative real-time reverse transcription PCR (qRT-PCR)

The cells were used when they reached ∼70% confluence. RNA was isolated using a SteadyPure RNA extraction kit, as per the manufacturer’s instructions (Accurate Biology, Changsha, China). The RNA was reverse transcribed to cDNA using ABScript Ⅲ RT Master Mix with RNA (1 μg) from each sample (ABclonal, Wuhan, China). The specific cDNAs were quantified using quantitative real-time PCR (qPCR) together with EvaGreen 2× qPCR MasterMix (Applied Biological Materials, Richmond, BC, Canada) and measuring the fluorescence intensity. Each reaction system was composed of 2 μL of cDNA, 5 μL of EvaGreen 2× qPCR MasterMix (Applied Biological Materials), 2.4 μL of DEPC (diethypyrocarbonate)-treated water (Sangon Biotech Co., Ltd. Shanghai, China), and 0.3 μL of 10 μM forward and reverse primer mix. Samples were amplified under the following cycling conditions: 95°C for 10 min, followed by 40 cycles of 95°C for 15 s and 60°C for 60 s. Cycle thresholds were calculated as the expression fold changes using the formula: 2^-△△CT^(Livak and Schmittgen, 2001). Data were analyzed using the Applied Biosystems 7500Real-Time PCR System (Applied Biosystems, Foster City, CA, United States). The Applied Biosystems QuantStudio 7 PRO Real-Time Fluorescence Quantitative PCR System runs were acquired and the results were analyzed using EXCEL (Microsoft Corp., Redmond, WA, United States). The primers used for qPCR, including those for the control *GAPDH* gene (encoding glyceraldehyde-3-phosphate dehydrogenase), were supplied by Sangon Biotech Co., Ltd. and are shown in the additional file (Supplementary Table S1). For the RNA-seq experiment, total RNA was extracted from H1299^vector^ and H1299^shEIF4G1^ (H1299 cells transfected with the empty vector or the vector expressing an shRNA targeting *EIF4G1*), followed by Novogene sequencing and data analysis (Novogene, Beijing, China).Gene Set Enrichment Analysis (GSEA) is a computational approach to determine if a pre-defined Gene Set can show a significant consistent difference between two biological states. The genes were ranked according to the degree of differential expression in the two samples, and then the predefined Gene Set were tested to see if they were enriched at the top or bottom of the list. Gene set enrichment analysis can include subtle expression changes. We use the local version of the GSEA analysis tool http://www.broadinstitute.org/gsea/index.jsp, KEGG and DisGeNET data sets were used for GSEA independently.

### Enzyme-linked immunosorbent assay (ELISA)

H1299 cells (1.6× 10^6^ cells per mL) were added to each well of a six-well plate. The medium was changed after 8 h. The supernatant was collected after 24 h and tested using an ELISA kit provided by ABclonal (Wuhan, China), following the manufacturer’s instructions.

### Western blotting analysis

Cells were used when they reached ∼70% confluence. Cells were washed twice with Dulbecco’s phosphate-buffered saline (DPBS; Corning), and then incubated for 30 min on ice in Radioimmunoprecipitation assay (RIPA) lysis buffer (Epizyme, Cambridge, MA, United States) with the addition of one percent protease phosphatase inhibitor (NCM Biotech, Newport, RI, United States). The cells were centrifuged, and the supernatant was collected and added to SDS loading buffer (Beyotime, Jiangsu, China), followed by boiling at 100°C for 10 min. After cooling, the samples were subjected to 7.5% SDS-polyacrylamide gel electrophoresis (Epizyme), followed by the electrophoretic transfer of the separated proteins to a polyvinylidene fluoride (PVDF) membrane (Millipore, Billerica, MA, United States). After blocking, the membranes were incubated with the following primary antibodies: Anti-EIF4G1, and anti-β-Actin (Cell Signaling Technologies (CST), Danvers, MA, United States; 1:1,000); anti-mitogen-activated protein kinase 14 (MAPK14 or p38), anti-phospho-p38, anti-rabbit IκBα, anti-phospho-IκBα, anti-JNK1/2/3, anti-phospho-JNK1/2/3, anti-ERK1/ERK2, anti-phospho-ERK1/ERK2, anti-TNFRSF10A, and anti-TNFRSF11B (ABclonal), followed by incubation with secondary antibodies comprising horseradish peroxidase (HRP)-labeled goat anti-rabbit IgG secondary antibody (Epizyme). The immunoreactive protein bands were visualized using a chemiluminescent solution (Thermo Fisher Scientific), and photographs were acquired using a fully-automated chemiluminescent imaging analysis system (Tanon Science & Technology CO., Shanghai, China).

### Macrophage chemotaxis

Macrophage migration assays were performed using Transwell chambers (Labselect, Hefei city, China) with an 8-μm pore size. Human monocytic leukemia cells, THP-1, were induced to differentiate into macrophages by treatment with 100 ng/mL phorbol 12-myristate-13-acetate (PMA; Sigma, St. Louis, MO, United States) in complete medium for 48 h. Then, 2 × 10^6^ cells per mL were added to the upper layer of the chamber. The H1299^vector^ group migrated for 24 h after the addition of H1299^vector^ conditioned medium (CM) to the lower chamber and the addition of different concentrations of human recombinant CXCL8 (ABclonal). Macrophages in the upper chamber of the H1299^shEIF4G1^ group migrated for 24 h after incubation with different concentrations of the CXCR1/2 inhibitor Navarixin (MK7123, #HY10198, MedChemExpress, Monmouth Junction, NJ, United States) for 24 h after 30 min of incubation at 37°C.

### Immunofluorescence assay

Cells were used for the assay when they reached ∼70% confluence. Cells were fixed using 4% paraformaldehyde and added with 0.25% TritonX-100, followed by 10% goat serum, and incubated at room temperature. After the goat serum was aspirated off, fresh goat serum containing anti-TNFRSF10A or anti-TNFRSF11B primary antibodies (ABclonal) was added to the cells, and incubated at room temperature for 2 h, followed by incubation with fluorescently-labeled secondary antibodies (Thermo Fisher Scientific) at room temperature and protected from light for 1 h. Finally, the cell nuclei were stained using 0.1% 4′,6-diamidino-2-phenylindole (DAPI) (Beyotime) for 10 min at room temperature and protected from light. Images were acquired under a microscope (Leica, Wetzlar, Germany).

### RNA interference

To construct *EIF4G1 s*table knockdown cell lines, cells were transfected with a Dharmacon (Lafayette, CO, United States) *EIF4G1*-shRNA, or a non-silencing (NS)-shRNA as a control.

### Cell survival assay

Cells were grown in 96-well plates with 10,000 cells per well. Different concentrations of SBI-756 (a water soluble inhibitor of EIF4G1; MedChemExpress) and TNFSF10 were added to the cells, which were incubated at 37°C, 5% CO_2_ and 95% humidity in an incubator for 24 h. Thereafter, 10 μL of Cell Counting Kit 8 (CCK-8) reagent (Share-bio, Hangzhou, China) was added and incubated for 2 h, after which the optical density (OD) values were measured at 450 nm in a microplate reader.

### NSCLC tissue samples

NSCLC tissues and their paracancerous control tissues were collected from 22 patients attending Shanghai Eastern Hospital. All experiments were approved by the Human Rights Committee of Shanghai Eastern Hospital.

The experimental protocol was established according to the ethical guidelines of the Helsinki Declaration and was approved by the Ethics Committee of the Shanghai East Hospital (No. 2019052). Written informed consent was obtained from individuals or their guardians.

### Bio-plex analysis

Concentrations of multiple cytokines in tissues were assayed after extraction of tissue proteins using a Bio-plex Prot Human Cytokine Grp I Panel 27-Plex kit (Bio-Rad, Hercules, CA, United States) following the supplier’s guidelines.

### Statistical analysis

The count data in this paper were described using examples and percentages, and the measured data were described using the mean ± standard deviation (mean ± SD). The statistical methods mainly used the χ^2^ test, *t*-test, and analysis of variance (ANOVA). *p* < 0.05 indicates that the difference is statistically significant, and *p* > 0.05 indicates that the difference is not statistically significant. Statistical analysis was performed using Stata 16 (Stata Corp., College Station, TX, United States) and GraphPad Prism 7 (GraphPad Inc., La Jolla, CA, United States). GraphPad Prism 7 and Adobe Photoshop 2022 (Adobe, San Jose, CA, United States) were used to plot the graphs.

## Results

### Downregulation of *EIF4G1* expression specifically increases CXCL8 expression

**FIGURE 1:**
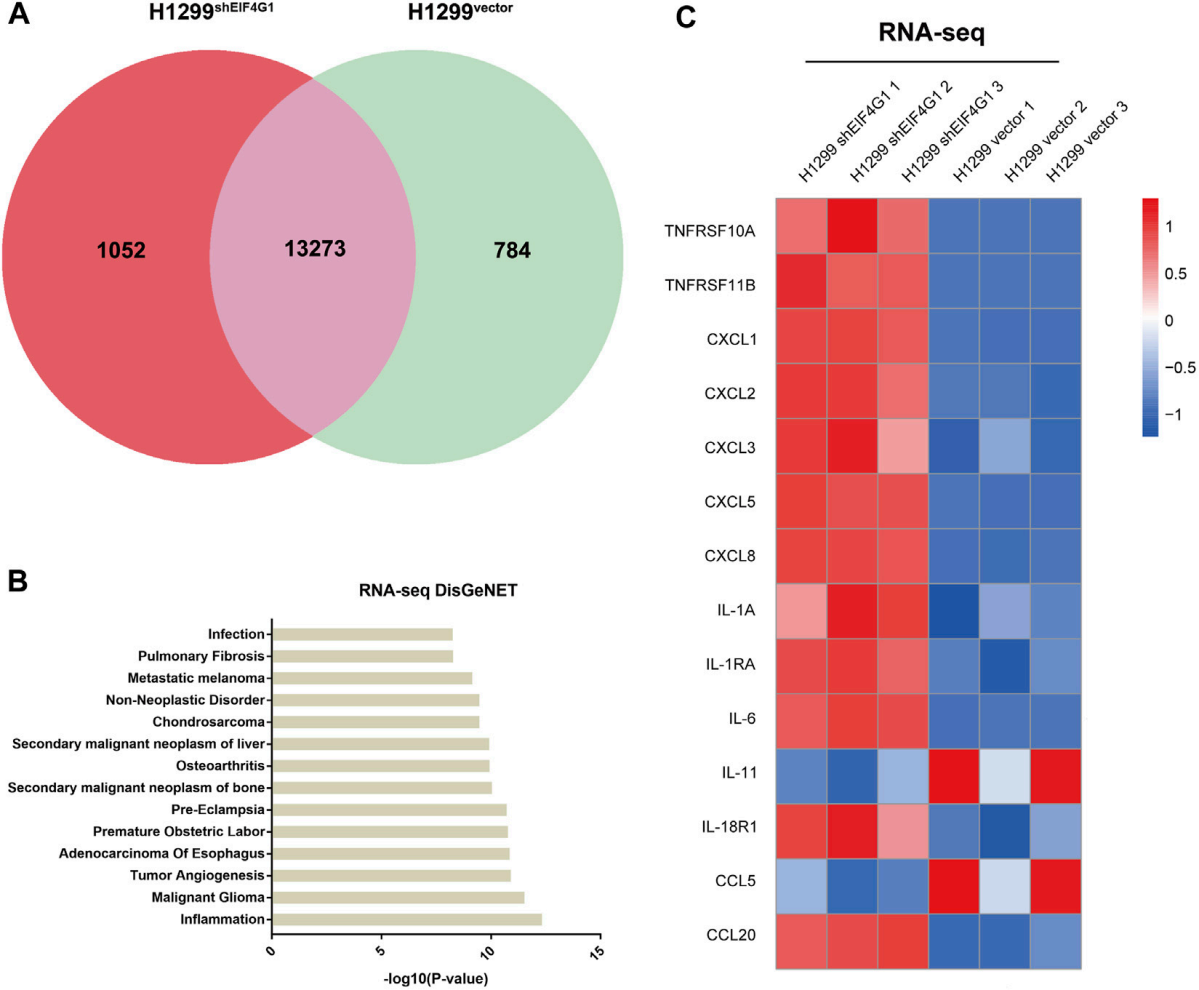
Downregulation of *EIF4G1* expression specifically increases CXCL8 expression **(A)** A co-expression Venn diagram showing the number of genes uniquely expressed in the two groups (H1299^vector^ and H1299^shEIF4G1^), the overlapping region shows the number of genes that were co-expressed between the two groups. **(B)** The DisGeNET database integrates human disease-related genes. DisGeNET pathways with corrected *p* values less than 0.05 were considered significantly enriched for differentially expressed genes. We used the clusterProfiler R package (3.8.1) to test the statistical enrichment of differentially expressed genes in the DisGeNET pathways. The 14 DisGeNET terms with the most significant differences between H1299^Vector^ and H1299^shEIF4G1^ in the RNA-seq data. **(C)** Heatmaps of the RNA-seq results showing the relative expression levels of selected genes involved in inflammation in the H1299^vector^ and H1299^shEIF4G1^ groups.

EIF4G1 is highly expressed in various types of tumors and affects the prognosis of patients with laryngeal squamous cell carcinoma (LSCC). It also regulates LSCC cell cycle protein D levels and cell proliferation through the protein kinase B (AKT)/mechanistic target of rapamycin kinase (mTOR) pathway (Jaiswal et al., 2019). To further explore how EIF4G1 functions in tumors and affects those downstream molecules, we performed RNA-seq analysis of H1299^vector^ and H1299^shEIF4G1^ cells. Quantitative analysis of the RNA-seq data identified 1,836 differentially expressed genes and 13,273 co-expressed genes between the samples from the H1299vector and H1299shEIF4G1 groups (Figure 1A).According to DisGeNET analysis, knockdown of *EIF4G1* had the greatest impact on inflammation (Figure 1B), and the KEGG results showed that the expression levels of a variety of inflammation-related cytokines was significantly upregulated in *EIF4G1* knockdown cells (Figure 1C). In this study, some common inflammation-related cytokines were selected to test their relative expression using qRT-PCR, and the mRNA levels of 84% of the inflammation-related factors were found to be higher in H1299^shEIF4G1^ cells relative to their levels in H1299^vector^ cells (Supplementary Figure S1A).

Clinical samples identified multiple inflammation-related cytokines that were upregulated in NSCLC tissues compared with those in the corresponding paraneoplastic tissues.

**FIGURE 2:**
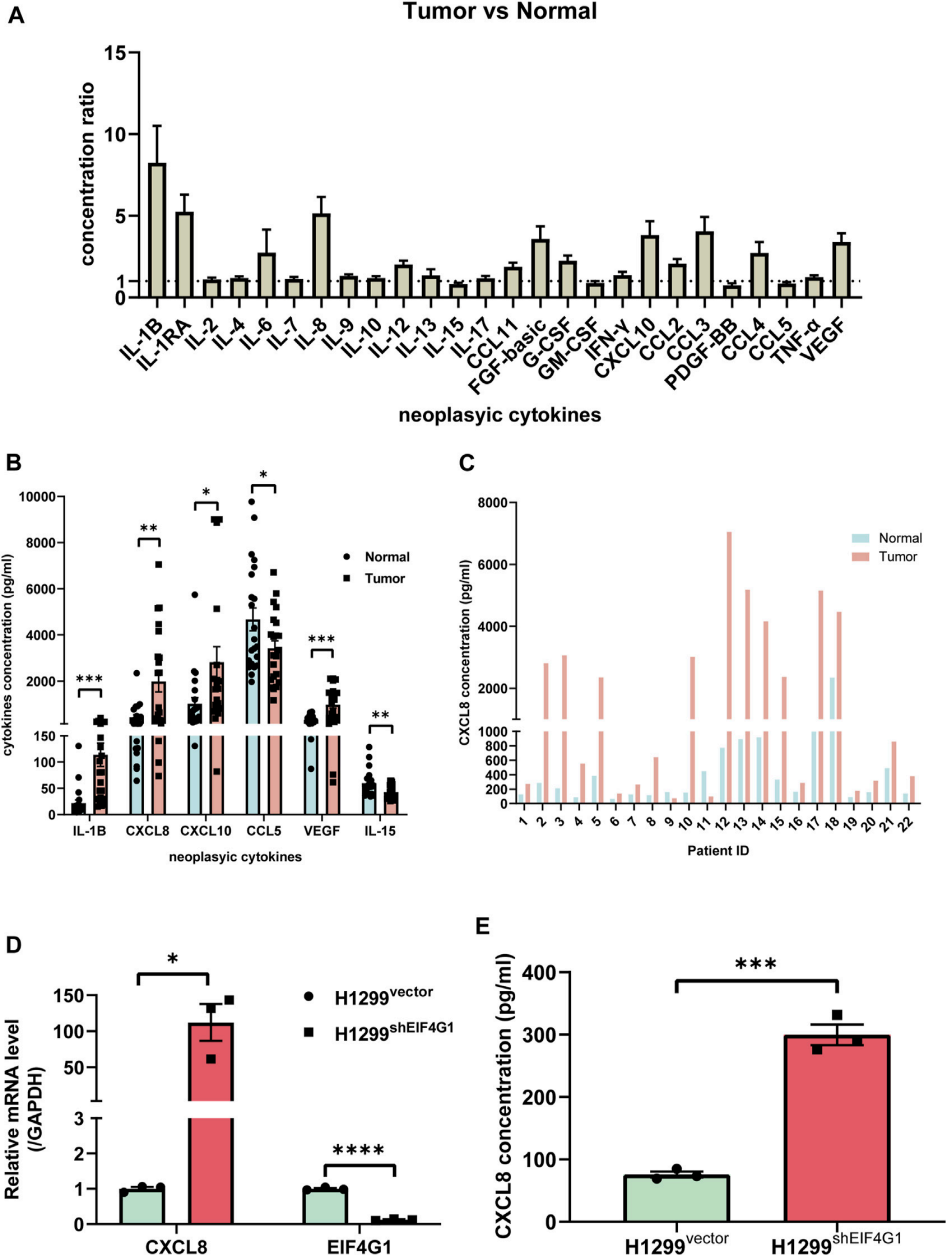
Clinical samples identified multiple inflammation-related cytokines that were upregulated in NSCLC tissues compared with those in the corresponding paraneoplastic tissues **(A)** Ratio of cytokine concentrations in non-small cell lung cancer tissues and their paracancerous control tissues from 22 patients with non-small cell lung cancer using a Bio-Plex Assay. **(B)** VEGF, CXCL8, CXCL10, IL-1b, bFGF, and CCL3 levels in non-small cell lung cancer tissues and their paracancerous control tissues from 22 patients with non-small cell lung cancer. **(C)** CXCL8 levels in lung cancer tissue samples from individual patients with non-small cell lung cancer and their paracancerous control tissues. **(D)** Relative mRNA levels of *CXCL8* and *EIF4G1* measured using qRT-PCR. **(E)** CXCL8 levels in supernatants of H1299^vector^ and H1299^shEIF4G1^ cells cultured for 24 h as determined using ELISA. Data are shown are mean ± SEM (n = 3). ****p < 0.0001, ***p < 0.001, **p < 0.01, and *p < 0.05.

Numerous studies have shown that inflammation is closely related to tumors, and that tumor cells regulate the inflammatory state by secreting various inflammatory mediators; however, inflammation is also involved in regulating cancer development, progression, and response to cancer therapy (Lee et al., 2021). To explore which inflammation-related cytokines are highly expressed in NSCLC, we used Bio-plex to examine 25 inflammation-related factors in cancer tissues from 22 patients with NSCLC and their control tissues. The results revealed that 18 inflammation-related cytokines were highly expressed in cancer tissues (Figure 2A). We investigated the relationship between NSCLC and the levels of various inflammation cytokines and found that interleukin (IL)1B (113.91 ± 21.59 vs. 21.87 ± 3.48), CXCL8 (1985.23 ± 438.51 vs. 430.08 ± 27.02), CXCL10 (2,819.79 ± 654.58 vs. 1,016.33 ± 70.82), and VEGF (981.78 ± 149.74 vs. 328.88 ± 42.79) were significantly elevated, and CCL15 (3,420.97 ± 317.86 vs. 4,672.99 ± 587.21) and IL15 (43.07 ± 2.71 vs. 59.73 ± 11.88) were significantly downregulated (Figure 2B). The results of transcriptome sequencing showed that low expression of EIF4G1 was associated with inflammation. Analysis of the expression of inflammation-related cytokines in lung cancer tissue samples from patients with NSCLC and their paracancerous control tissues, showed that a large number of inflammation-related cytokines were highly expressed in NSCLC tissues, including CXCL8, whose results were in line with those of transcriptome sequencing. Figure 2C shows the expression of CXCL8 in cancer tissues and its paracancerous tissues in each NSCLC patient. The correlation between CXCL8 and patient age, sex, tumor-node-metastasis (TNM) stage, and tumor grade was also analyzed; however, no statistical significance was found (Supplementary Table S2). Analysis using qRT-PCR and ELISA verified that knockdown of *EIF4G1* led to significantly high expression of CXCL8 (Figures 2D, E).

### EIF4G1 regulates CXCL8 by modulating TNFRSF10A expression

**FIGURE 3:**
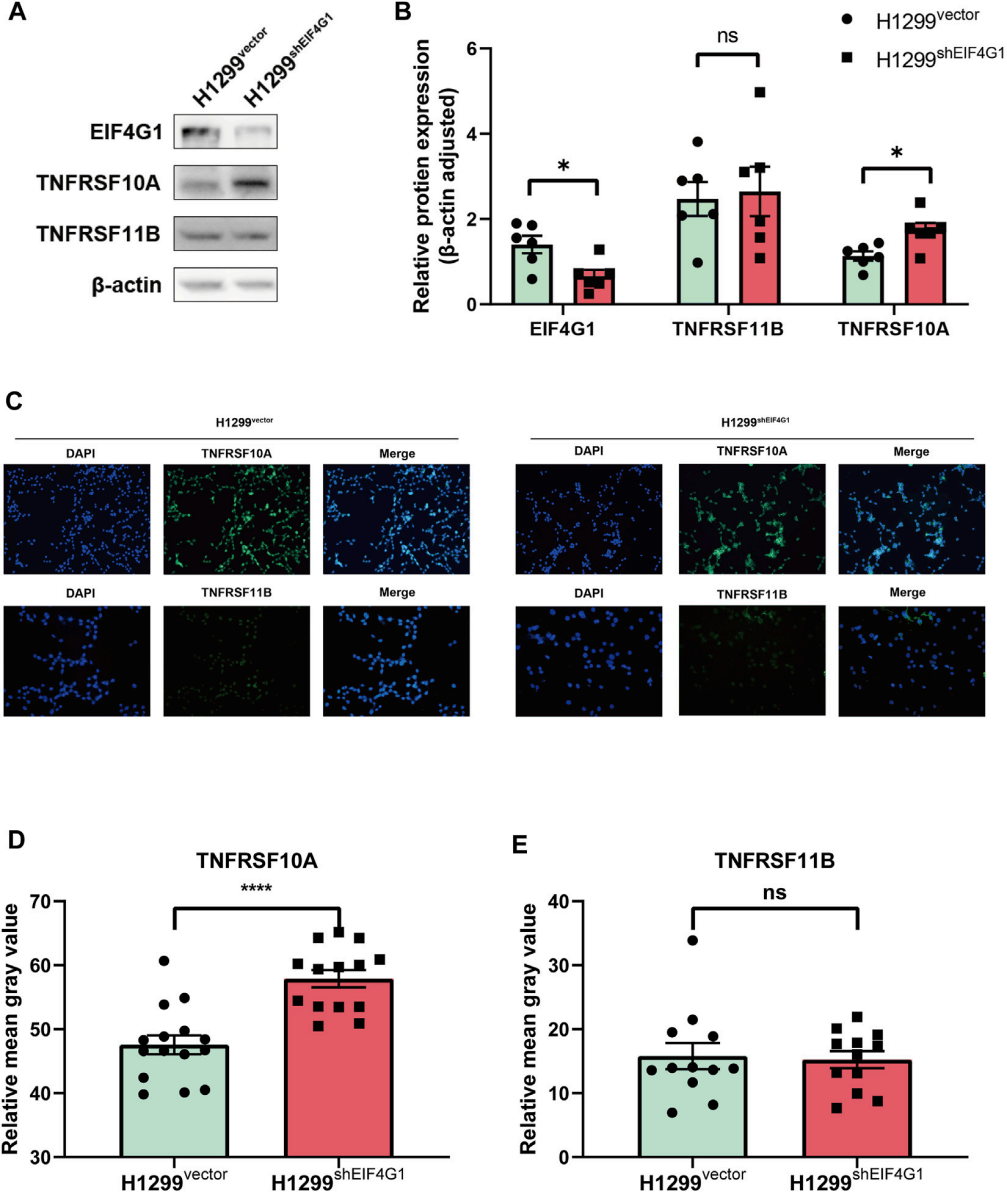
EIF4G1 regulates CXCL8 by modulating TNFRSF10A expression **(A,B)** Protein levels of EIF4G1, TNFRSF10A, and TNFRSF11B were examined **(A)** and quantified **(B)** using western blotting. **(C–E)** Cell surface TNFRSF10A and TNFRSF11B levels were detected **(C)** and measured **(D,E)** using an immunofluorescence assay. Data are expressed as the mean ± SEM from three independent experiments. ****p < 0.0001 and *p < 0.05. ns: no significance.

A previous study showed that TNFSF10 stimulation of pancreatic ductal adenocarcinoma (PDAC) cells induced the secretion of CXCL8, and the expression of CXCL8 correlated with the concentration of TNFSF10 added to the cells (Legler et al., 2018). The results of transcriptome sequencing (Figure 1C) showed that *TNFRSF10A* and *TNFRSF11B*, encoding the specific receptors of TNFSF10, were significantly highly expressed at the mRNA level in H1299^shEIF4G1^ cells. This was verified using qRT-PCR, and the amplification curves showed that the H1299^vector^ cells hardly expressed *TNFRSF10A* and *TNFRSF11B* (Supplementary Figures S1B–D). Interestingly, when examining the expression of both proteins using Western blotting, we observed that the TNFRSF10A level was upregulated when *EIF4G1* was knocked down, whereas TNFRSF11B expression did not change significantly (Figures 3A, B). This was also confirmed by immunofluorescence analysis (Figures 3C–E).

### TNFRSF10A regulates downstream inflammatory MAPK and NFκB pathways affecting CXCL8 expression

**FIGURE 4:**
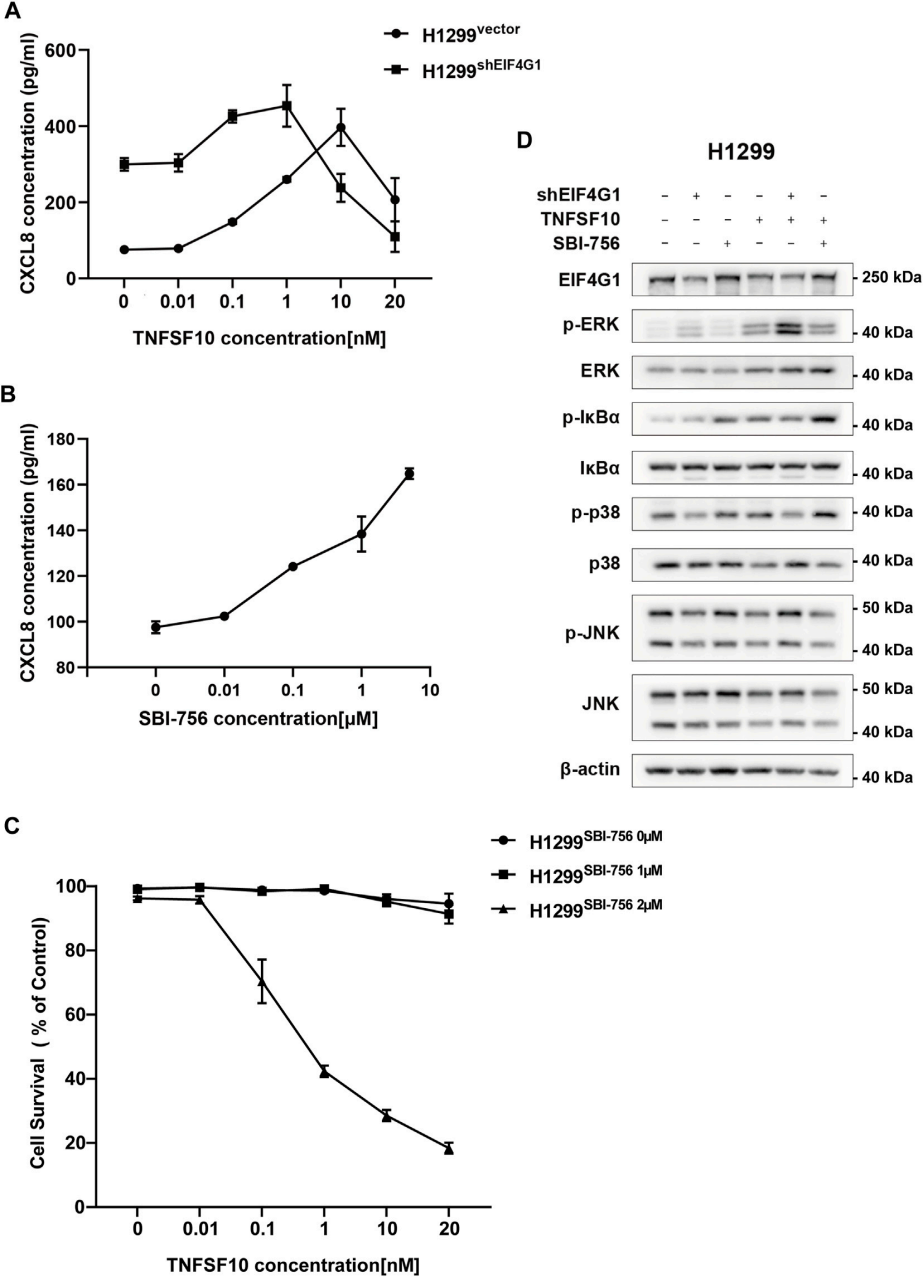
TNFRSF10A regulates downstream inflammatory MAPK and NFκB pathways affecting CXCL8 expression **(A)** H1299^vector^ and H1299^shEIF4G1^ cells were treated with the indicated concentrations of TNFSF10 for 24 h. The CXCL8 levels in the cell culture supernatants was determined using ELISA. Data are shown as the means ± SEM (n = 3). **(B)** H1299 cells were treated with the indicated concentrations of SBI-756 for 24 h. CXCL8 levels in cell culture supernatants were determined using ELISA. Data are shown as the means ± SEM (n = 3). **(C)** H1299cells were treated with SBI-756 (0, 1, and 2 μM) and TNFSF10 (0, 0.01, 0.1, 1, 10, and 20 nM) for 24 h. Cell viability was determined using a CCK8 assay. **(D)** Cells were treated with TNFSF10 (1 nM) and/or SBI-756 (1 μM) for 24 h. Western blotting for ERK1/2, IκBα, p38, JNK1/2/3, p-ERK1/2, p-IκBα, p-p38, and p-JNK1/2/3 is shown.

H1299^vector^ and H1299^shEIF4G1^ cells were incubated for 24 h in culture medium with the addition of recombinant TNFSF10 at concentrations of 0, 0.01, 0.1, 1, 10, and 20 nM. The concentration of CXCL8 in the supernatant of the cell culture media was detected using ELISA. It was clear that, with the addition of increasing amounts ofTNFSF10, the H1299^shEIF4G1^ cells showed the highest level of CXCL8 secretion at a TNFSF10 concentration of 1 nM, while H1299^vector^ cells showed the highest level of CXCL8 secretion at a TNFSF10 concentration of 10 nM (Figure 4A). SBI-756 is an inhibitor of EIF4G1, capable of disrupting the EIF4F complex. The concentration of CXCL8 in the supernatant of the cell culture medium gradually increased with the addition of increasing concentrations of SBI-756 in H1299 cells (Figure 4B).TNFSF10 has the ability to promote necrotic cell death in tumor cells; therefore, we compared the effects of adding 0, 1, and 2 μM SBI-756 to H1299 cells while adding 0, 0.01, 0.1, 1, 10, and 20 nM of recombinant TNFSF10 and tested cell survival. We observed that EIF4G1 inhibition or knockdown increased the sensitivity of the cells to TNFSF10 (Figure 4C). Functions of the TNFR superfamily show that their excitation activates related downstream inflammatory pathways and mediates tumor apoptosis (Ashkenazi, 2008). Therefore, we analyzed the phosphorylation levels (activation) of JNK1/2/3, p38, and ERK1/2 of the MAPK pathway and IκBα of the NFκB pathway in H1299^vector^, H1299^shEIF4G1^, and H1299^vector SBI−756^ cells after incubation for 24 h with 1 nM recombinant TNFSF10, respectively. The levels of phospho-p38, phospho-ERK1/2, and phospho-IκBα increased upon the addition of TNFSF10, and knockdown of *EIF4G1* resulted in a significant increase in ERK1/2 phosphorylation, whereas the addition of SBI-756 to the cells caused upregulation of IκBα phosphorylation, with the end result being an increase in the level of CXCL8 (Figure 4D).

### *EIF4G1* knockdown in H1299 cells promotes CXCL8-induced macrophage chemotaxis toward tumor cells

**FIGURE 5:**
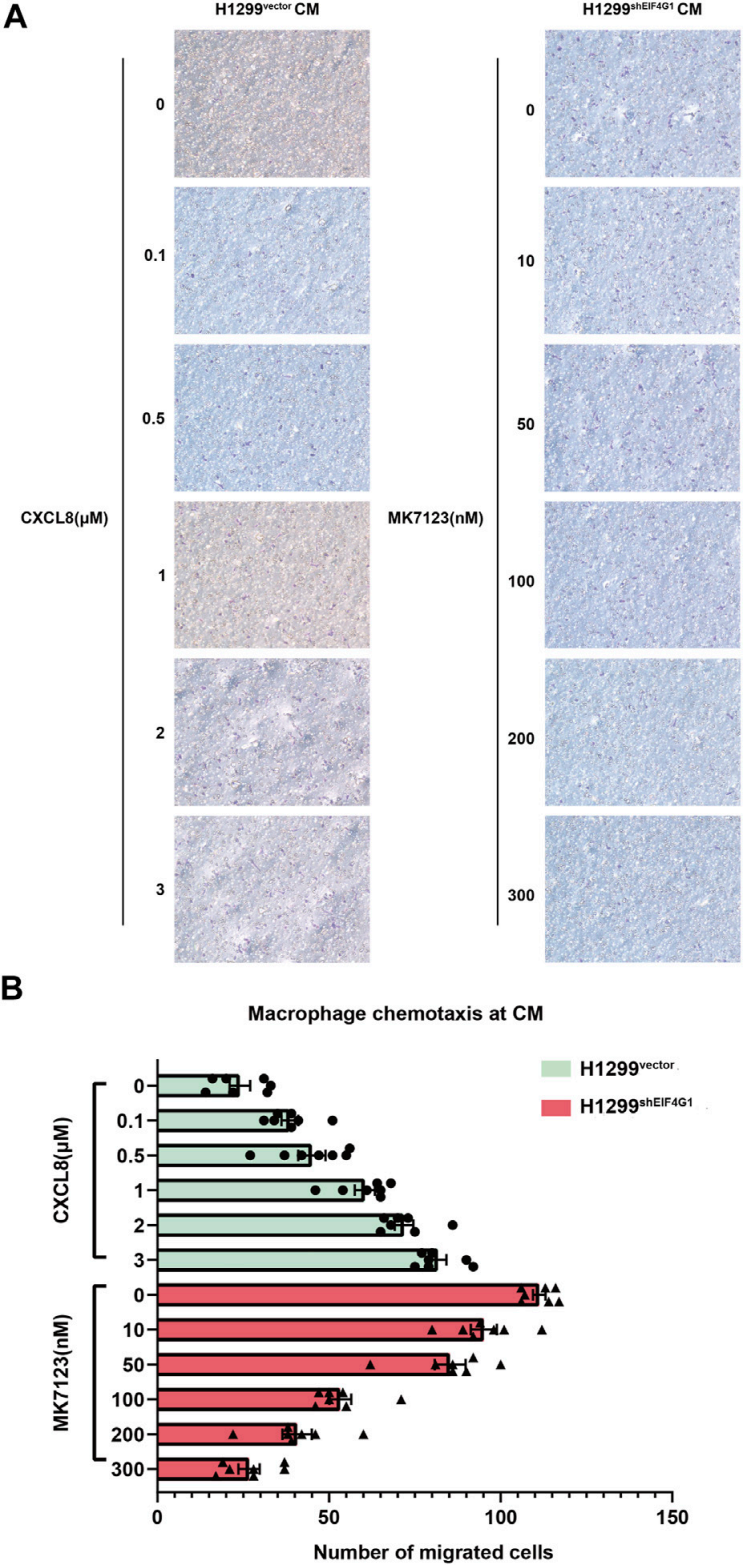
*EIF4G1* knockdown in H1299 cells promotes CXCL8-induced macrophage chemotaxis toward tumor cells **(A,B)** CM from H1299^vector^ and H1299^shEIF4G1^ cells was prepared by adding complete medium and incubating for 24 h. Recombinant CXCL8 was added at concentrations of 0, 0.1, 0.5, 1, 2, and 3 μM to the CM of H1299^vector^ cells in the lower layer of the Transwell chambers, and THP-1 cells were added to the upper layer after 24 h of PMA induction in the upper layer of macrophages. Macrophages in the upper layer of another set of Transwell chambers after 24 h of THP-1 induction by PMA were first pretreated with 30 min of MK7123, a small molecule inhibitor of the CXCL8 receptor. CXCR1/CXCR2 were added to macrophages at concentrations of 0, 10, 50, 100, 200, and 300 nM, and the H1299^shEIF4G1^ CM was added to the lower chamber for the macrophage chemotaxis experiments.

Macrophages are usually the most abundant immune population present in the TME and are a major component of the immune infiltration in solid tumors. As a chemokine, CXCL8 has the ability to induce targeted chemotaxis of specific immune cells (Mantovani et al., 2002).To verify that EIF4G1 ultimately regulates CXCL8 secretion by affecting TNFRSF10A expression, we tested the ability of H1299^vector^ and H1299^shEIF4G1^ cells to promote CXCL8-induced macrophage chemotaxis using Transwell assays. We prepared H1299^vector^ and H1299^shEIF4G1^CM by culturing the cells in complete medium for 24 h. We added recombinant CXCL8 n at 0, 0.1, 0.5, 1, 2, and 3 μM to the CM of H1299^vector^. To the upper macrophage layer of the CM of H1299^shEIF4G1^, we added MK7123 at concentrations of 0, 10, 50, 100, 200, and 300 nM. MK7123 is a small molecule inhibitor of CXCL8 receptors CXCR1/CXCR2 on macrophages. We observed a gradual increase in the number of macrophages that underwent chemotaxis with the addition of recombinant CXCL8 at elevated concentrations in the CM of H1299^vector^, and a gradual decrease in the number of macrophages that underwent chemotaxis with the addition of MK7123 at elevated concentrations in the upper macrophage layer of the CM of H1299^shEIF4G1^. This suggests that *EIF4G1* knockdown in the H1299 cell line affects the chemotaxis of macrophages towards tumor cells by regulating CXCL8 (Figures 5A, B).

## Discussion

The translation initiation factor, EIF4G1, is highly expressed in a variety of tumors, and its ability to regulate the expression of numerous proteins has an important impact on tumor proliferation, migration, secretion of cytokines, and tumor angiogenesis (Fan et al., 2010; Hsieh et al., 2012; Robichaud et al., 2015). Previously, our group found that EIF4G1 expression is significantly higher in NSCLC tissues than in paraneoplastic tissues and affects patient prognosis, such that high expression of EIF4G1 in tumor tissues predicts shorter patient survival (Bai et al., 2023). The present study further explored the functions of EIF4G1 in tumors.

A study showed that the addition of TNFSF10 to PDAC cells resulted in changes in CXCL8 secretion (Legler et al., 2018). According to the analysis of the transcriptome sequencing data, the TNFSF10 receptor genes, *TNFRSF10A* and *TNFRSF11B*, were significantly highly expressed in H1299^shEIF4G1^ cells. However, when the protein levels of TNFRSF10A and TNFRSF11B were analyzed, it was found that TNFRSF10A was significantly overexpressed in H1299^shEIF4G1^ cells, but there was no significant difference in TNFRSF11B levels between H1299^vector^ and H1299^shEIF4G1^ cells, which might have been caused by the fact that P53 is naturally absent from the H1299 cell line and P53 is responsible for the translation of TNFRSF11B. During the preliminary phase of the experiment, we used both A549 and H1299 cell lines after RNA-seq. Surprisingly, the results were not the same. Although knocking down *EIF4G1* significantly increased the CXCL8 expression level in the H1299 cell line, adding different concentrations of TNFSF10 did not significantly affect CXCL8 expression in A549 cells. This difference might be due to the fact that A549 is a p53 wild-type cell line, while H1299 is homogeneously partially deficient for the p53 protein. The difference in p53 protein levels between A549 (wild-type) and H1299 (partially deficient) might explain the observed results. Literature reports indicate that p53 regulates NFκB in melanoma, affecting the expression of various cytokines, including CXCL8 (Pandya et al., 2022). Our group is currently investigating the impact of p53 on CXCL8 expression in NSCLC cells. Thus, only the protein level of TNFRSF10A changed when the mRNA levels of TNFRSF10A and TNFRSF11B were increased. To verify whether increased secretion of CXCL8 in lung cancer cells is caused by increased expression of the receptor for TNFSF10, TNFRSF10A, after knockdown of *EIF4G1*, the protein concentration of CXCL8 was detected in response to different concentrations of TNFSF10 in H1299^vector^ and H1299^shEIF4G1^ cells, respectively. We found that the concentration of added TNFSF10 correlated positively with the CXCL8 secretion level, but it was consistently higher in the *EIF4G1* knockdown group than in the null control group, and the added amount of TNFSF10 in the null control group needed to be elevated by nearly 100-fold to induce the same CXCL8 secretion level as that in the *EIF4G1* knockdown group. TNFSF10 is a member of the TNF family of tumor necrosis factors and has the function of promoting tumor cell necrosis (Montinaro and Walczak, 2023). The combined use of SBI-756, an inhibitor of EIF4G1, and TNFSF10 in H1299 cells revealed that SBI-756 promoted TNFSF10-induced necrosis of tumor cells at lower concentrations, which suggested that EIF4G1-augmented TNFRSF10A expression significantly increased the sensitivity of lung cancer cells to TNFSF10.

A study by Favaro et al. reported that in NSCLC, glucose withdrawal, or treatment with TNFSF10 or TNFα, enhanced CXCL8 secretion. Moreover, in A549 cells, TNFRSF10A and TNFRSF11B activated CXCL8 production by regulating FADD, caspase-8, RIPK1, and TRADD, and the downstream ERK, MAPK, and MEK signaling pathways (Favaro et al., 2022). Therefore, we tested whether TNFSF10 can also activate the MAPK signaling pathway to mediate the synthesis of CXCL8 by activating its own TNFR receptor on the cell surface in H1299 cells, and whether it is related to EIF4G1. We examined the levels of key phosphorylated molecules in the MAPK signaling pathway in cells incubated for 24 h with H1299^vector^, H1299^shEIF4G1^, and H1299^vector^ treated with 1 nM SBI-756 and with 2nM TNFSF10, respectively. We observed that ERK was highly phosphorylated relative to the null control both before and after the addition of TNFSF10 when *EIF4G1* was knocked down, which is the main reason for the upregulation of CXCL8 expression after *EIF4G1* knockdown. The phosphorylation of IκBα, which is a binding inhibitor of the transcription factor p65 in the NFκB pathway, leads to the further addition of ubiquitylated tags, which are recognized by the proteasome for degradation, so that more transcription factors can enter the nucleus to participate in transcription (Capece et al., 2022). Phosphorylation of p38 was not significantly affected by changes in EIF4G1 or by the addition of TNFSF10. JNK had no significant downstream effect without TNFSF10, whereas phosphorylation of JNK in the *EIF4G1* knockdown group after the addition of TNFSF10 was significantly elevated, and thus was also an important contributor to the greater synthesis of CXCL8. Combined with the previous findings, it seemed that lung cancer cells became more sensitive to TNFSF10 by increasing the expression of TNFRSF10A, which led to an increased activation of the MAPK signaling pathway and the movement of various types of downstream transcription factors into the nucleus, which was ultimately manifested by upregulated protein and mRNA levels of CXCL8.

CXCL8 has been reported to function to direct the chemotaxis of various types of immune cells, such as neutrophils, macrophages, endothelial cells, among which, macrophages are associated with increased inflammation (Vandercappellen et al., 2008; Nagarsheth et al., 2017). The upregulation of CXCL8 expression caused by *EIF4G1* knockdown promoted macrophage chemotaxis, and the addition of MK7123, a receptor inhibitor of CXCL8, inhibited this process in a dose-dependent manner, suggesting that the chemotaxis of macrophages toward lung cancer cells is mainly dependent on CXCL8. *In vivo*, it is possible that macrophages are more likely undergo chemotaxis toward tumor tissues with low EIF4G1 expression, thereby promoting inflammatory responses in tumor tissues. This study explored the chemotaxis of tumor cells toward tumor-associated macrophages (TAMs) in the TME (Li M. et al., 2023a). TAMs exhibit phenotypic heterogeneity in different patients, as well as in different disease stages in patients with the same tumor (Li et al., 2022; Pittet et al., 2022). This heterogeneity reflects the ability of TAMs to respond to environmental stimuli, leading to phenotypic polarization into types ranging from pro-inflammatory (M1-like) to anti-inflammatory (M2-like) types (Yang et al., 2020; Li et al., 2022). Based on our previous research, we found that the expression of EIF4G1 changes during tumor development. Future studies will investigate whether chemotactic macrophages entering the TME are affected by varying levels of EIF4G1 expression (Bai et al., 2023).

In the present study, we found that decreased expression of EIF4G1 correlated highly with inflammation, with the most significant changes occurring in CXCL8 expression. EIF4G1 activates the MAPK signaling pathway by regulating the expression of TNFRSF10A, thereby controlling the secretion of CXCL8. In addition, we found that EIF4G1 exerts a pro-inflammatory effect by modulating CXCL8 to affect the chemotaxis of macrophages toward tumor cells, a view supported by a previous report on CXCL8 (Shi et al., 2021). This study was the first to link EIF4G1 to inflammation. TNFSF10, a small molecule drug that selectively induces apoptosis, is not as hepatotoxic as traditional chemotherapeutic agents; however, some tumor cells are not sensitive to TNFSF10 (Law et al., 2022; Luiz-Ferreira et al., 2023; Pimentel et al., 2023). Herein, we found that regulating the level of EIF4G1 can affect the sensitivity of tumor cells to TNFSF10 by altering the expression of TNFSF10 receptors, which has good clinical prospects.

## Data Availability

The raw data supporting the conclusion of this article will be made available by the authors, without undue reservation.
