# Supplementary material for: Knockdown of EIF4G1 in NSCLC induces CXCL8 secretion
Source: Front Pharmacol. 2024 Feb 9;15:1346383. doi: 10.3389/fphar.2024.1346383 (PMC10884238; doi:10.3389/fphar.2024.1346383)
Supplement: Supplementary file 1 [file Table1.DOCX]

Supplemental Table S1: qRT-PCR primers used in this study

| CXCL8 Forward primer | AGTTTTTGAAGAGGGCTGAGA |
| --- | --- |
| CXCL8 Reverse primer | TGCTTGAAGTTTCACTGGCATC |
| TNFRSF10A Forward primer | CTTTGGTTGTTCCGTTGCTGT |
| TNFRSF10A Reverse primer | GGTCCCCTCCACAACCTGAG |
| TNFSF11B Forward primer | TCCAAGCCCCTGAGGTTTCC |
| TNFSF11B Reverse primer | AGATGTCCAGAAACACGAGCG |
| EIF4G1 Forward primer | CTCTTCATCTTTGTACGGCATG |
| EIF4G1 Reverse primer | CCTTGGTAGTACTGAGCAGTAG |
| ANG Forward primer | ATTCTTCCTCCTGGGAGCCTG |
| ANG Reverse primer | GGGTCAGGAAGTGTGTGTACC |
| CCL2 Forward primer | TCAAACTGAAGCTCGCACTCT |
| CCL2 Reverse primer | GGCATTGATTGCATCTGGC |
| CCL5 Forward primer | CCCCATATTCCTCGGACACC |
| CCL5 Reverse primer | CATCCTTGACCTGTGGACGA |
| CXCL1 Forward primer | CCCAAACCGAAGTCATAGCCA |
| CXCL1 Reverse primer | TTCTTAACTATGGGGGATGCAG |
| CXCL2 Forward primer | GAAAGCTTGTCTCAACCCCG |
| CXCL2 Reverse primer | TGGTCAGTTGGATTTGCCATTTT |
| CXCL3 Forward primer | AAAAGATACTGAACAAGGGGAGCA |
| CXCL3 Reverse primer | CTCTGGTAAGGGCAGGGACC |
| CXCL5 Forward primer | CTGCAAGTGTTCGCCATAGG |
| CXCL5 Reverse primer | TCCTTGTTTCCACCGTCCAA |
| EGF Forward primer | TCTACTTGTGTGGGTCCTGC |
| EGF Reverse primer | TCACTGAGACACCAGCATCC |
| FGF2 Forward primer | GCGACCCTCACATCAAGCTA |
| FGF2 Reverse primer | AGCCAGGTAACGGTTAGCAC |
| FIGF Forward primer | TGCTGGAACAGAAGACCACTC |
| FIGF Reverse primer | ACAGACACACTCGCAACGAT |
| IFNG Forward primer | GAGTGTGGAGACCATCAAGGA |
| IFNG Reverse primer | TGGACATTCAAGTCAGTTACCGAA |
| IGF1 Forward primer | TTGCTTCATTATTCCTGCTAACC |
| IGF1 Reverse primer | TGGTGTGCATCTTCACCTTCA |
| IL6 Forward primer | CCTTCTCCACAAGCGCCTTC |
| IL6 Reverse primer | GGAAGGCAGCAGGCAACA |
| LEP Forward primer | GATTCTTGTGGCTTTGGCCC |
| LEP Reverse primer | AGGAGACTGACTGCGTGTGT |
| PDGFB Forward primer | GGAGTCGGCATGAATCGCT |
| PDGFB Reverse primer | AATGGGGTCCCCCTCGG |
| PGF Forward primer | AATGTCACCATGCAGCTCCT |
| PGF Reverse primer | ACGTGCTGAGAGAACGTCAG |
| TGFB1 Forward primer | GGAAATTGAGGGCTTTCGCC |
| TGFB1 Reverse primer | CCGGTAGTGAACCCGTTGAT |
| THPO Forward primer | CTTGGAACCCAGCTTCCTCC |
| THPO Reverse primer | CAAGCATCAGGAAACGCACC |
| TIMP1 Forward primer | ATTCCGACCTCGTCATCAGG |
| TIMP1 Reverse primer | GCATCCCCTAAGGCTTGGAA |
| TIMP2 Forward primer | TAGTGATCAGGGCCAAAGCG |
| TIMP2 Reverse primer | CTCAGGCCCTTTGAACATCTTT |
| TPO Forward primer | CAGTGCAGTTGGCTGAGAAG |
| TPO Reverse primer | CTCCTCAGGCTTTCCCCAAA |
| VEGFA Forward primer | ACAAATGTGAATGCAGACCAAA |
| VEGFA Reverse primer | ACCAACGTACACGCTCCAG |
| CCL1 Forward primer | CTTCACCAGGCTCATCAAAGC |
| CCL1 Reverse primer | AGAAGGGTACCTGCATGCTCTT |
| CCL11 Forward primer | TCCAACATGAAGGTCTCCGC |
| CCL11 Reverse primer | GTGGTTGGGACAGAAGCTGG |
| CCL24 Forward primer | CCTGTTACCTCCGGGTCCTT |
| CCL24 Reverse primer | GAGCCCGTAGGGATGATGTG |
| CSF2 Forward primer | CTGGAGCTGTACAAGCAGGG |
| CSF2 Reverse primer | ACAGGAAGTTTCCGGGGTTG |
| CSF3 Forward primer | AAGCTGGTGAGTGAGTGTGC |
| CSF3 Reverse primer | ATGGAGTTGGCTCAAGCAGC |
| IL10 Forward primer | TTGCTGGAGGACTTTAAGGGTT |
| IL10 Reverse primer | TCACATGCGCCTTGATGTCT |
| IL11 Forward primer | GGGGACATGAACTGTGTTTGC |
| IL11 Reverse primer | GGGCGACAGCTGTATCTGG |
| IL12A Forward primer | AGCACAGTGGAGGCCTGTTT |
| IL12A Reverse primer | GCCAGGCAACTCCCATTAGTT |
| IL13 Forward primer | TTGCACAGACCAAGGCCC |
| IL13 Reverse primer | AGCTGTCAGGTTGATGCTCC |
| IL1A Forward primer | GCGTTTGAGTCAGCAAAGAAGT |
| IL1A Reverse primer | CAGAGACAGATGATCAATGGAGGA |
| IL1B Forward primer | TTCGAGGCACAAGGCACAA |
| IL1B Reverse primer | TGGCTGCTTCAGACACTTGAG |
| IL2 Forward primer | CAACTGGAGCATTTACTGCTGG |
| IL2 Reverse primer | TCAGTTCTGTGGCCTTCTTGG |
| IL3 Forward primer | CTTCGAAGGCCAAACCTGGA |
| IL3 Reverse primer | GATTGGATGTCGCGTGGGT |
| IL7 Forward primer | CGCAGACCATGTTCCATGTTTC |
| IL7 Reverse primer | AGATGATGCTACTGGCAACAGA |

Supplemental Table S2:

Correlations between CXCL8 expression and clinic-pathological features of 22 NSCLC patients in Bio-Plex

| Variables | N | CXCL8 expression | | P value |
| --- | --- | --- | --- | --- |
|  |  | Mean (pg/ml) | SEM (pg/ml) |  |
| Sample |  |  |  | 0.0016 |
| Normal | 22 | 430.08 | 27.02 |  |
| Tumor | 22 | 1985.23 | 438.51 |  |
|  |  |  |  |  |
| Age |  |  |  | 0.6164 |
| < 55 | 6 | 1842.84 | 543.95 |  |
| ≥ 55 | 16 | 2364.94 | 669.46 |  |
|  |  |  |  |  |
| Sex |  |  |  | 0.4683 |
| Male | 15 | 1756.07 | 504.12 |  |
| Female | 7 | 2476.28 | 825.77 |  |
|  |  |  |  |  |
| Tumor size |  |  |  | 0.7060 |
| T1–T2 | 12 | 1825.15 | 496.40 |  |
| T3–T4 | 10 | 2177.32 | 754.38 |  |
|  |  |  |  |  |
| Lymph node |  |  |  | - |
| N− | 1 | 263.96 | 0.00 |  |
| N+ | 21 | 2067.20 | 451.67 |  |
|  |  |  |  |  |
| Metastasis |  |  |  | - |
| M0 | 2 | 2708.10 | 1728.27 |  |
| M1 | 20 | 1912.94 | 447.13 |  |
|  |  |  |  |  |
| Grade |  |  |  | 0.7322 |
| 1~ 2 | 10 | 1810.92 | 704.16 |  |
| 3 | 12 | 2130.50 | 545.99 |  |

Supplemental figure：

**Fig. S1**

(**A**) mRNA expression levels of 31 inflammation-related cytokines in H1299^shEIF4G1^ relative to those in H1299^vector^. (**B**) Relative mRNA levels of *TNFRSF10A* and *TNFRSF11B* as measured using qRT-PCR. *, significant (p < 0.05) versus the respective control. (**C**,**D**) Amplification profiles of *TNFRSF10A* and *TNFRSF11B*, respectively.
