# Supplementary figures and images for: Knockdown of EIF4G1 in NSCLC induces CXCL8 secretion
Source: Front Pharmacol. 2024 Feb 9;15:1346383. doi: 10.3389/fphar.2024.1346383 (PMC10884238; doi:10.3389/fphar.2024.1346383)

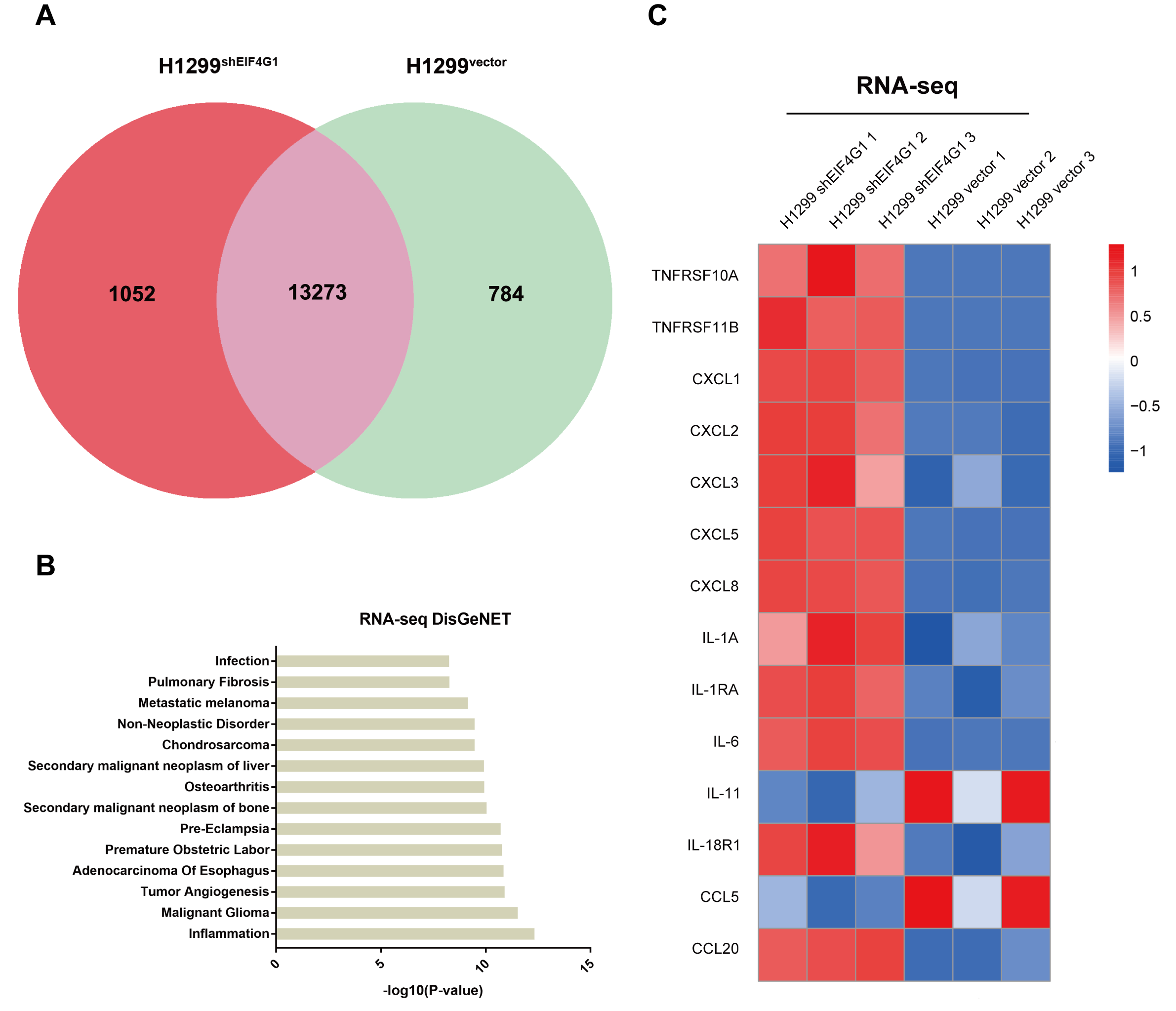

Supplement: Supplementary file 2 [file DataSheet1.ZIP › tiff/figure 1.tif]

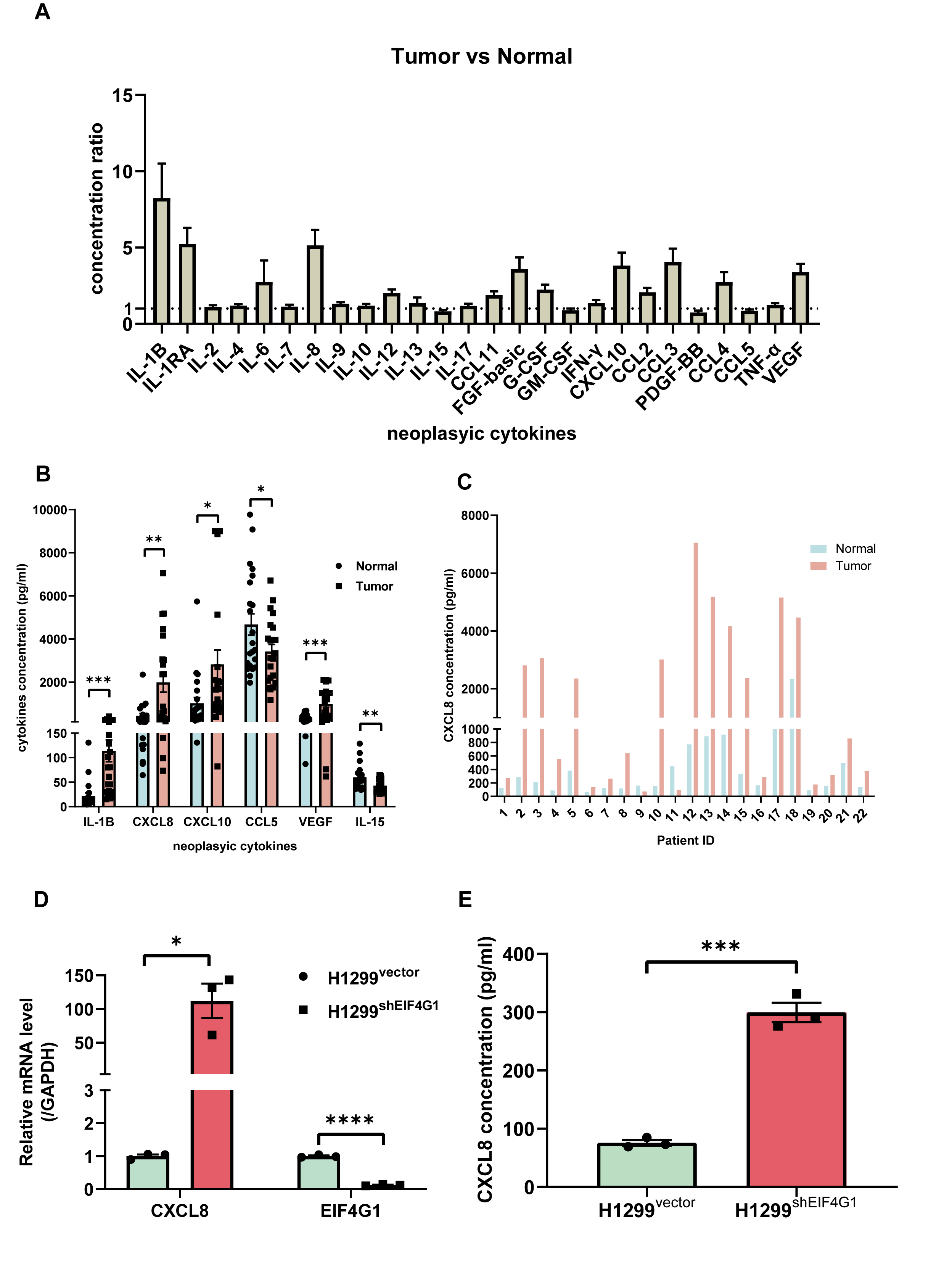

Supplement: Supplementary file 2 [file DataSheet1.ZIP › tiff/figure 2.tif]

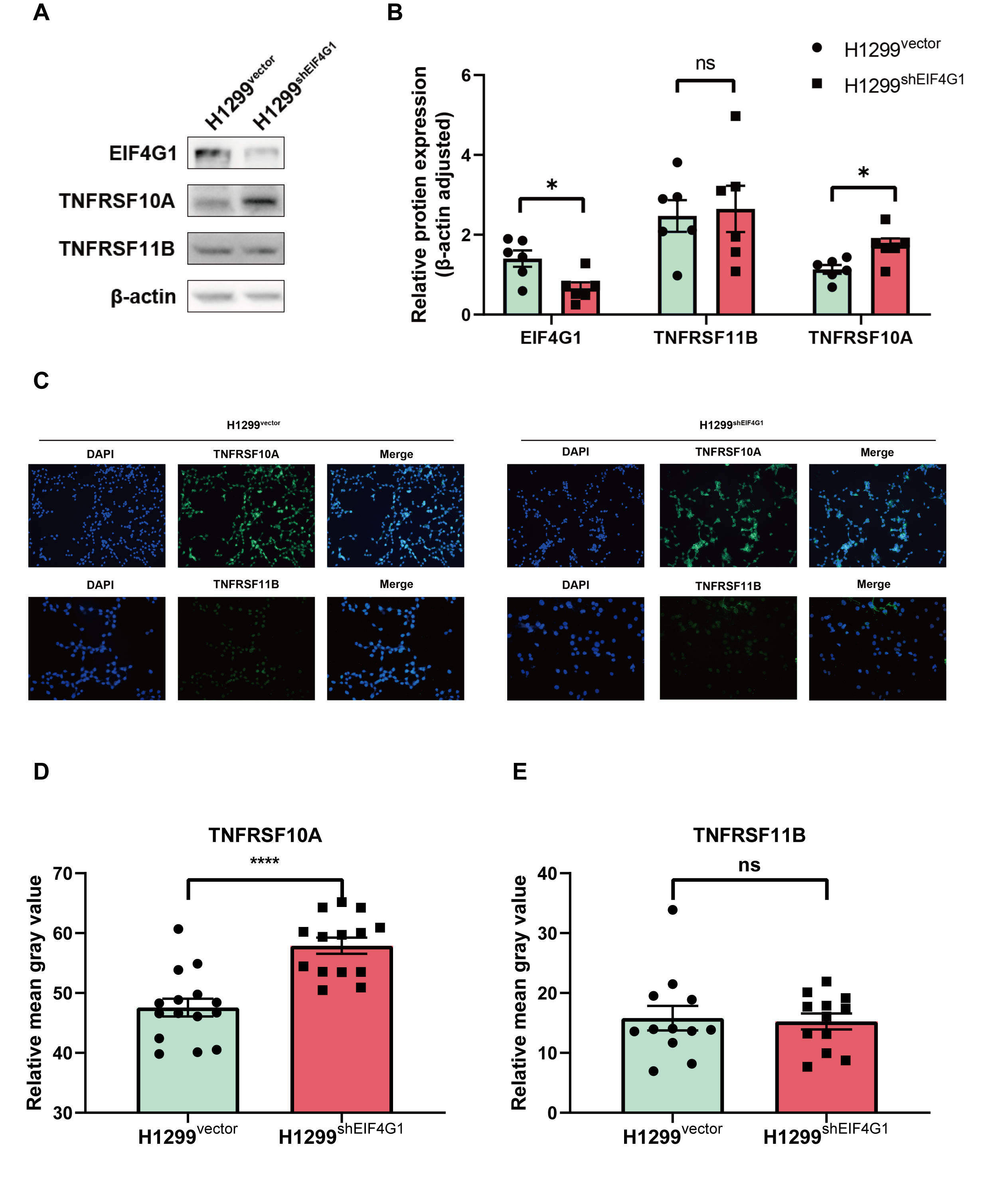

Supplement: Supplementary file 2 [file DataSheet1.ZIP › tiff/figure 3.tif]

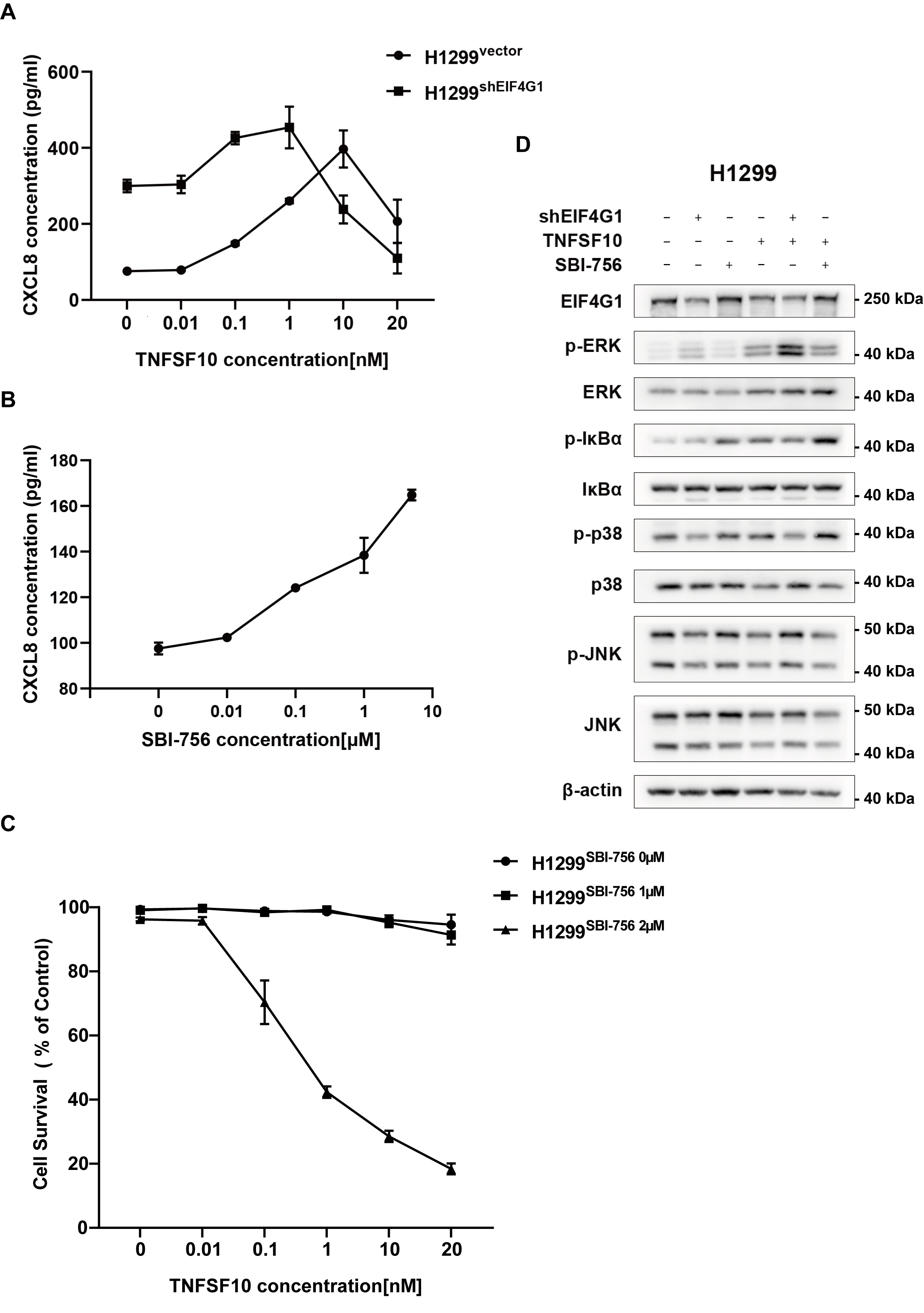

Supplement: Supplementary file 2 [file DataSheet1.ZIP › tiff/figure 4.tif]

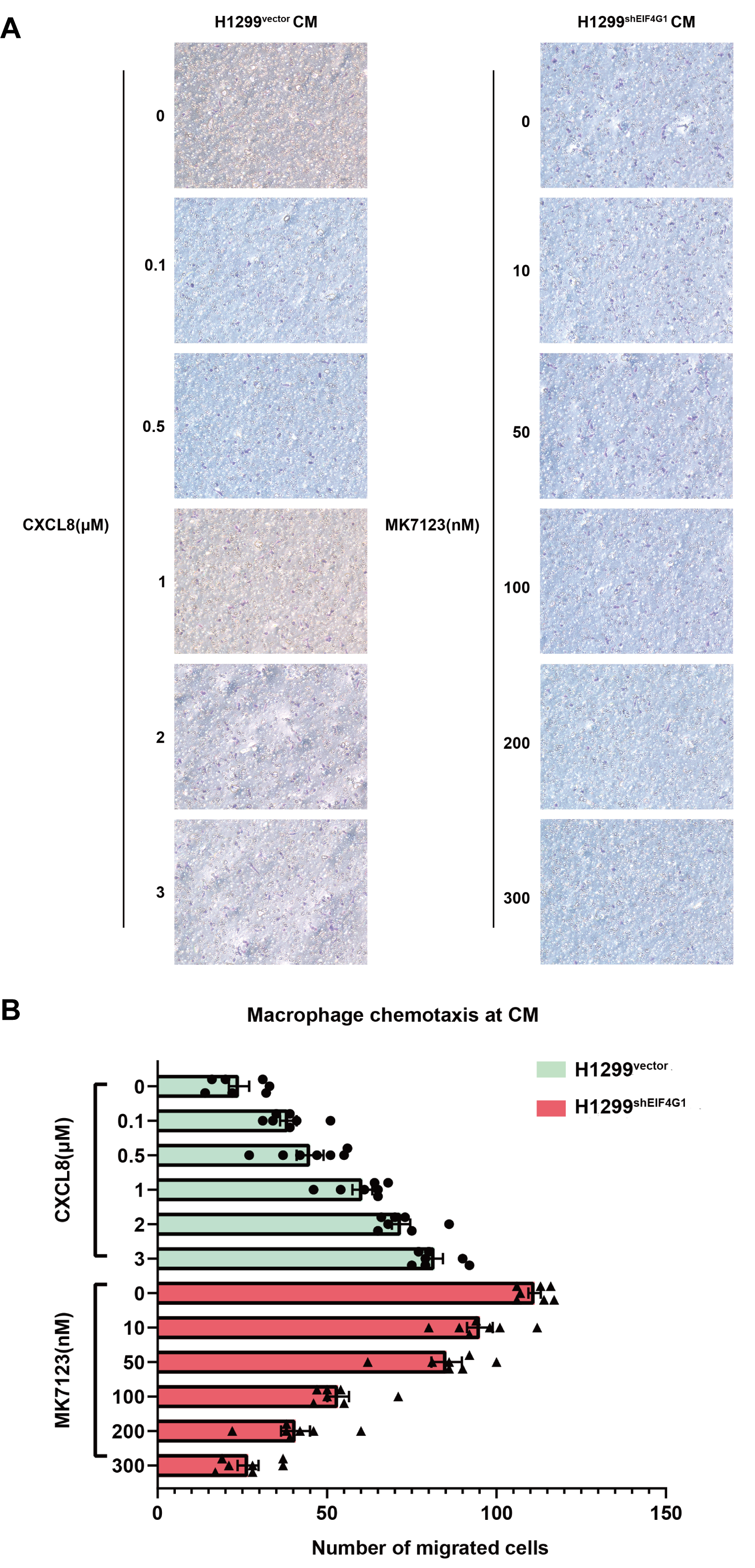

Supplement: Supplementary file 2 [file DataSheet1.ZIP › tiff/figure 5.tif]

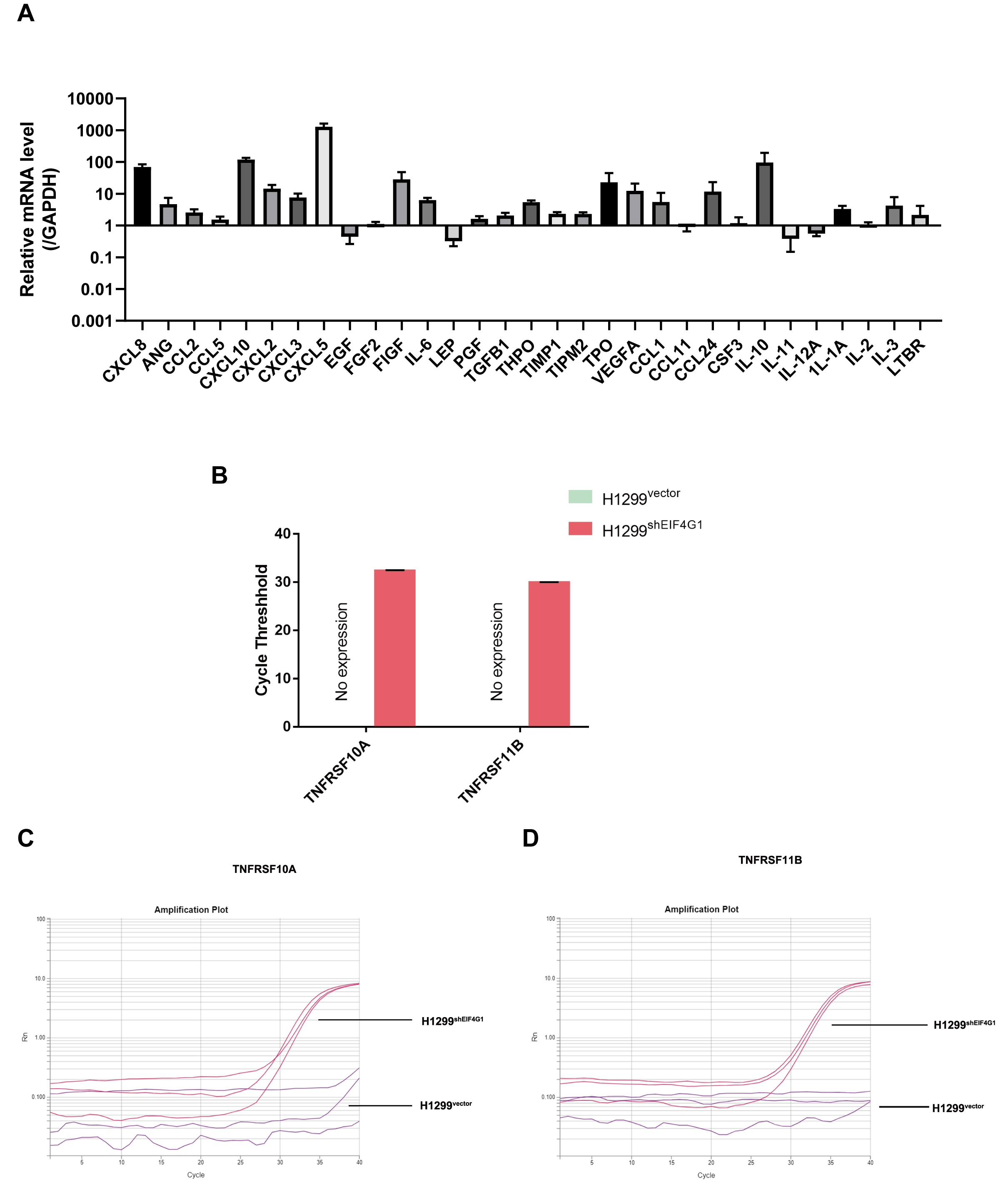

Supplement: Supplementary file 2 [file DataSheet1.ZIP › tiff/figure S1.tif]

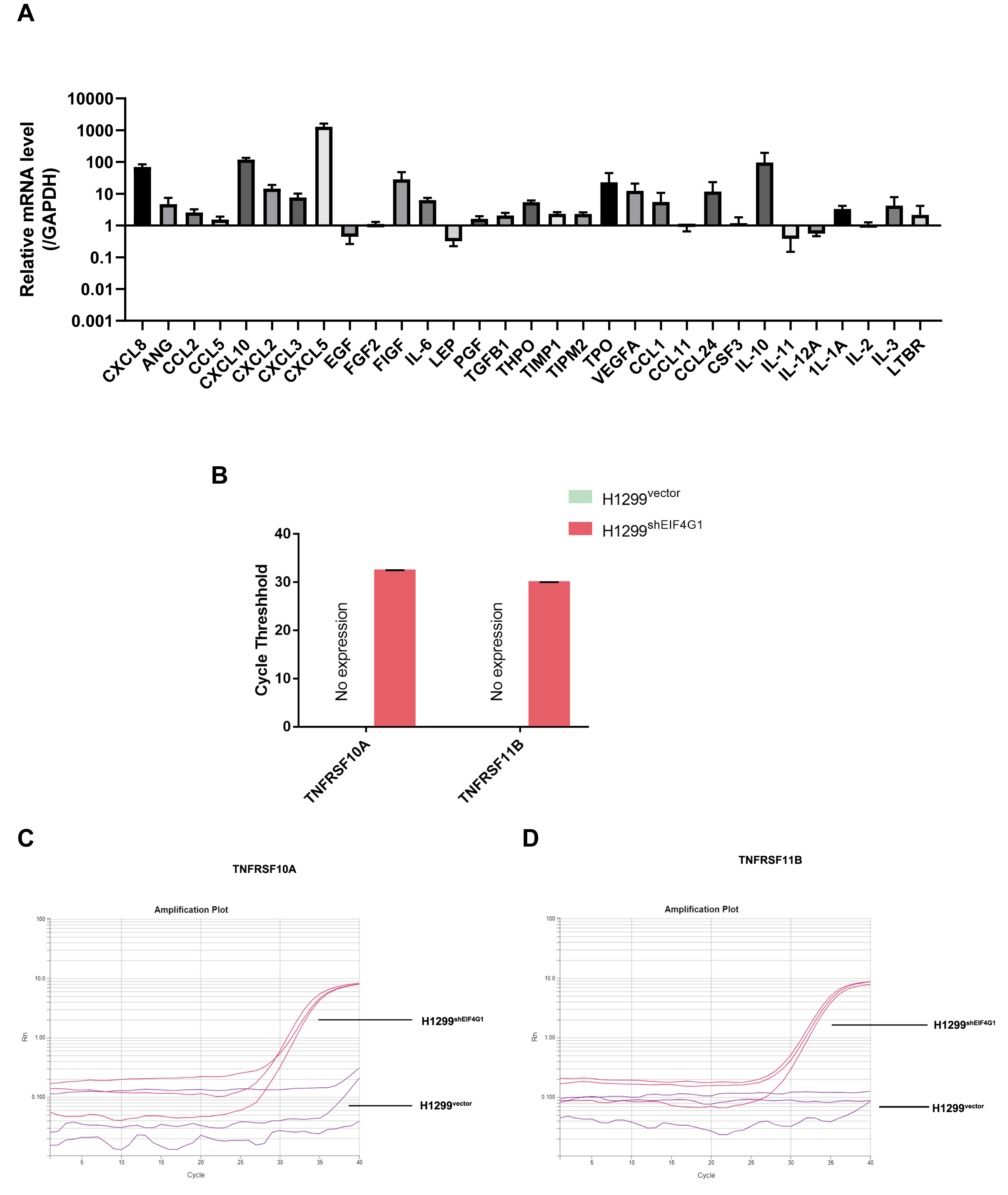

Supplement: Supplementary file 3 [file Image1.TIF]
